# A one-year prospective study of colonization with antimicrobial-resistant organisms on admission to a Vietnamese intensive care unit

**DOI:** 10.1371/journal.pone.0184847

**Published:** 2017-09-14

**Authors:** Duong Bich Thuy, James Campbell, Nguyen Van Minh Hoang, Truong Thi Thuy Trinh, Ha Thi Hai Duong, Nguyen Chi Hieu, Nguyen Hoang Anh Duy, Nguyen Van Hao, Stephen Baker, Guy E. Thwaites, Nguyen Van Vinh Chau, C. Louise Thwaites

**Affiliations:** 1 Hospital for Tropical Diseases, Wellcome Trust Major Overseas Programmes, Oxford University Clinical Research Unit (OUCRU), Ho Chi Minh City, Vietnam; 2 Adult Intensive Care Unit, Hospital for Tropical Diseases, Ho Chi Minh City, Vietnam; 3 Centre for Tropical Medicine and Global Health, Nuffield Department of Clinical Medicine, Oxford University, Oxford, United Kingdom; 4 Department of Infectious Diseases, University of Medicine and Pharmacy, Ho Chi Minh City, Vietnam; 5 Department of Medicine, Cambridge University, Cambridge, United Kingdom; 6 Board of Directors, Hospital for Tropical Diseases, Ho Chi Minh City, Vietnam; University of California San Francisco, UNITED STATES

## Abstract

There is a paucity of data regarding initial bacterial colonization on admission to Intensive Care Units (ICUs) in low and middle-income countries (LMICs). Patients admitted to ICUs in LMICs are at high-risk of subsequent infection with antimicrobial-resistant organisms (AROs). We conducted a prospective, observational study at the Hospital for Tropical Diseases in Ho Chi Minh City, Vietnam from November 2014 to January 2016 to assess the colonization and antimicrobial susceptibility of *Staphylococcus aureus*, *Escherichia coli*, *Klebsiella* spp., *Pseudomonas* spp. and *Acinetobacter* spp. among adult patients within 48 hours of ICU admission. We found the admission colonization prevalence (with at least one of the identified organisms) was 93.7% (785/838) and that of AROs was 63.1% (529/838). The colonization frequency with AROs among patients admitted from the community was comparable to those transferred from other hospitals (62.2% vs 63.8%). *Staphylococcus aureus* was the most commonly isolated bacteria from nasal swabs (13.1%, 110/838) and the methicillin-resistant *Staphylococcus aureus* nasal colonization prevalence was 8.6% (72/838). We isolated *Escherichia coli* from rectal swabs from almost all enrolled patients (88.3%, 740/838) and 52.1% (437/838) of patients were colonized by extended spectrum β-lactamase producing *Escherichia coli*. Notably, *Klebsiella pneumoniae* was the most frequently isolated bacteria from the tracheal swabs (11.8%, 18/153). Vietnamese ICU patients have a high rate of colonization with AROs and are thus at risk of subsequent infections with these organisms if good infection control practices are not in place.

## Introduction

Leaders in world health have described antimicrobial-resistant organisms (AROs) as “nightmare bacteria” that account for a substantial number of excess deaths, and catastrophic healthcare spending [1]. The impact of AROs is suggested to be far more serious in low and middle-income countries (LMICs) than in well-resourced countries, where unregulated antimicrobial use, and poor infection control practices may result in increased numbers of infections related to AROs [2].

Infection with AROs is a major problem faced by Intensive Care Units (ICUs) located in LMICs. Progression from colonization to infection has been well established for ICU patients in well-resourced settings with comparably low rates of AROs [3–9]. However, little is known about initial bacterial colonization on admission to ICUs and subsequent infections in LMICs. The reason behind this lack of data is multifactorial but is partly associated with a lack of routine screening, which is rarely available or affordable. However, in such locations routine screening is vital due to the circulation of AROs in the community and increased likelihood of transmission within the ICUs due to poor infection control. To address this knowledge gap in bacterial colonization on a ICU in a LMIC, we conducted a one-year prospective study to assess the initial colonization and antimicrobial susceptibility profiles of *Staphylococcus aureus (S*. *aureus)*, *Escherichia coli (E*. *coli)*, *Klebsiella* spp. (including *K*. *pneumoniae)*, *Pseudomonas* spp. (including *P*. *aeruginosa))* and *Acinetobacter* spp. (including *A*. *baumannii)* on admission to the Adult ICU at the Hospital for Tropical Diseases (HTD) in Ho Chi Minh City (HCMC), Vietnam.

## Materials and methods

### Setting

This prospective, observational study was conducted in the 20-bed Adult ICU of the HTD in HCMC, Vietnam. HTD is a tertiary referral hospital for infectious diseases serving Southern Vietnam. The study was reviewed and approved by the Ethics Committee of the Hospital for Tropical Diseases in Ho Chi Minh City, Vietnam and the Oxford Tropical Research Ethics Committee (OxTREC), United Kingdom.

### Study population and procedure

All patients aged ≥ 15 years admitted to the Adult ICU between 10^th^ November 2014 and 14^th^ January 2016 were eligible for entry to the study. Written informed consent were obtained from the participants or their representatives. All patients whom were readmitted to Adult ICU of the HTD within 90 days from discharge were excluded. All eligible patients had nasal swab, rectal swab, and/or endotracheal aspirate (ETA, in case of intubation or tracheostomy) taken within 48 hours of ICU admission for screening *S*. *aureus*, *E*. *coli*, *Klebsiella* spp., *Pseudomonas* spp. and *Acinetobacter* spp..

### Microbiological methods

Nasal and endotracheal specimens were cultured on Blood agar (BioMerioux) and MacConkey (BioMerioux) to specifically isolate *S*. *aureus*, *E*. *coli*, *Klebsiella* spp., *Pseudomonas* spp. and *Acinetobacter* spp.. An experienced microbiologist selected probable Staphylococcal colonies from blood agar plates inoculated with the patient swabs, and checked for catalase and coagulase, then cultured on CHROMagar plus checked for methicillin resistance using cefotaxime and oxacillin disc diffusion, and re-checked on the matrix assisted laser desorption/ionization time-of-flight mass spectrometry (MALDITOF, Bruker).

Xylose Lysine Deoxcholate agar (BioMerioux) and MacConkey agar (BioMerioux) were used to culture rectal swabs for Gram negative bacteria, and identification of *E*. *coli*, *Klebsiella* spp., *Pseudomonas* spp. and *Acinetobacter* spp. was confirmed by MALDITOF. Antimicrobial susceptibility testing was conducted by the Kirby/Bauer disc diffusion method and interpreted using the Clinical and Laboratory Standards Institute (CLSI) guidelines 2015. Appropriate organisms were screened for specific antimicrobial-resistant activity including extended spectrum β-lactamase (ESBL) using CHROMagar ESBL, and the double disc diffusion test, AmpC β-lactamase (AmpC) using CHROMagar AmpC, and carbapenemases using CHROMagar KPC.

### Definition of colonization

For the purposes of this investigation, admission colonization was assessed as a positive nasal, rectal and/or endotracheal sample for *S*. *aureus*, *E*. *coli*, *Klebsiella* spp., *Pseudomonas* spp. and/or *Acinetobacter* spp. within 48 hours of ICU admission. AROs colonization was assessed as a positive culture from nasal, rectal and/or endotracheal sample of MRSA, 3^rd^-generation cephalosporin (3^rd^ CPS)-resistant *E*. *coli*, 3^rd^ CPS-resistant *Klebsiella* spp., ceftazidime-resistant *Pseudomonas* spp., carbapenem-resistant *E*. *coli*, carbapenem-resistant *Klebsiella* spp., carbapenem-resistant *Pseudomonas* spp. and/or carbapenem-resistant *Acinetobacter* spp..

### Statistical analysis

Descriptive analyses of the endpoints were performed, consisting of frequency (percentage) for categorical data, and median (95% confidence interval– 95% CI or interquartile range—IQR) for continuous data using R statistical software (R Core Team (2013). R: A language and environment for statistical computing. R Foundation for Statistical Computing, Vienna, Austria). Comparisons of percentage were performed using χ^2^ or Fisher’s exact tests. Comparisons of means were performed using Student’s t test or nonparametric tests, such as the Kruskal-Wallis test. P-values < 0.05 (two-sided) were considered statistically significant.

## Results

### General patient characteristics

During the one-year study period, 838 of 873 (96.0%) patients admitted the Adult ICU of the HTD met the inclusion criteria and were enrolled in the study. An additional 35 (4.0%) patients, who were readmitted to the Adult ICU within 90 days and were excluded. Patient characteristics (age, sex, patient origin, history of antimicrobial therapy within 90 days and 24 hours prior to ICU admission) and clinical information (Charlson Comorbidity Index and APACHE II score) were collected using a standardized form on the day of enrollment. The baseline demographic characteristics are shown in Table 1.

**Table 1:** General patient characteristics upon ICU admission.

Characteristics	All patients (N = 838)
**Age (yr)—median (IQR)**	43 (29–59)
< 60—n (%)	634 (75.7)
≥ 60—n (%)	204 (24.3)
**Sex—n (%)**	
Male	497 (59.3)
Female	341 (40.7)
**Origin of patients—n (%)**	
From the community	333 (39.7)
From other hospitals	419 (50.0)
From another general ward at HTD	86 (10.3)
**History of previous antimicrobial therapy within 90 days**	58 (6.9)
**History of previous antimicrobial therapy within 24 hours**	181 (21.6)
**Charlson Comorbidity Index—median (IQR)**	0 (0–1)
No comorbidity (0)—n (%)	512 (61.1)
Mild (1–2)—n (%)	166 (19.8)
Moderate (3–4)—n (%)	108 (12.9)
Severe (≥ 5)—n (%)	52 (6.2)
**APACHE II score—median (IQR)**	8 (4–14)
Mild (< 5)—n (%)	240 (28.6)
Moderate (5–12)–n (%)	330 (39.4)
Severe (> 12)–n (%)	268 (32.0)

### Admission colonization status

**Fig 1:**
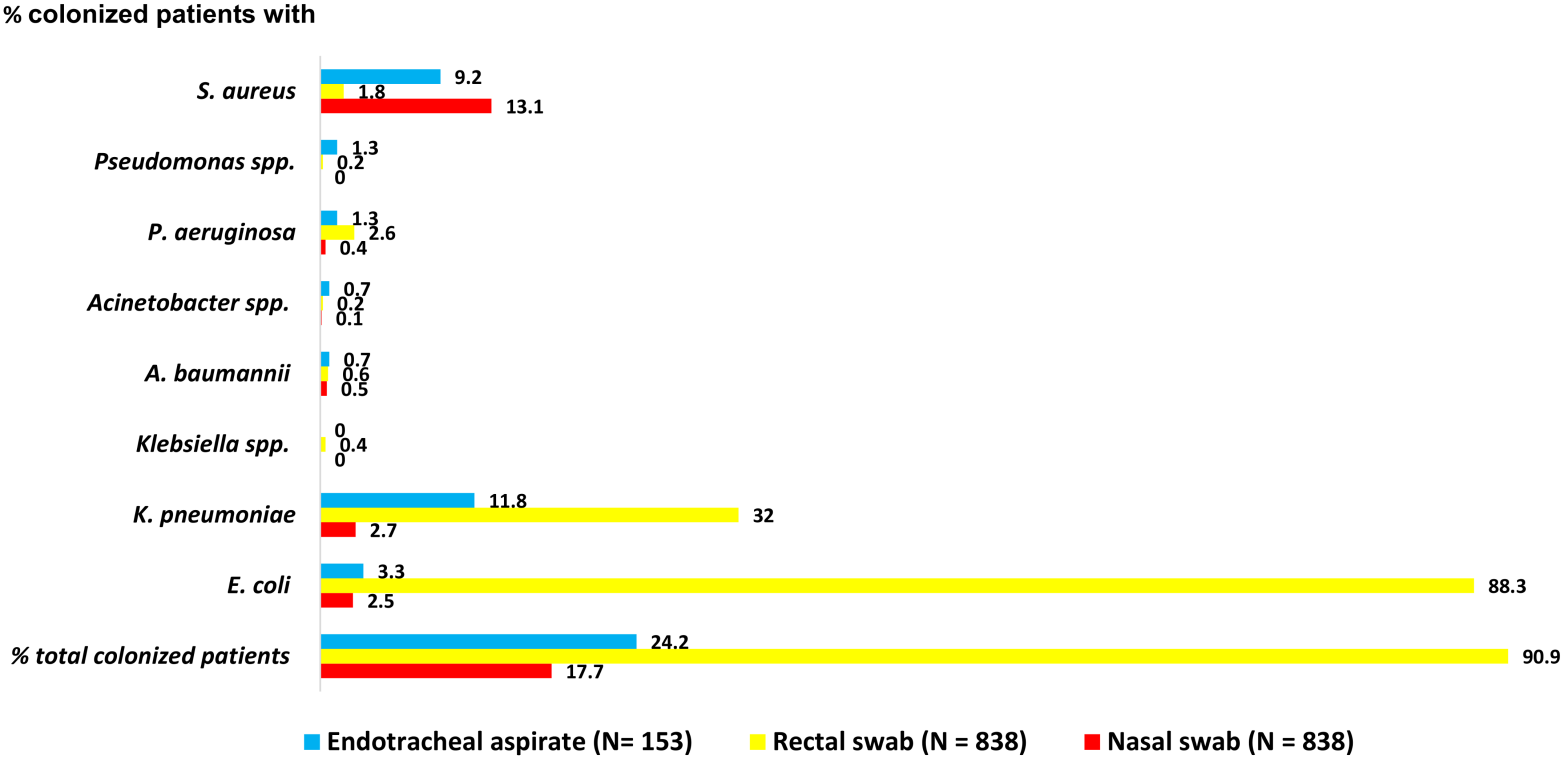
Admission colonization status of 838 enrolled patients.

A total of 1,829 sampling swabs were taken from the 838 enrolled patients within 48 hours of ICU admission: 838 were nasal swabs, 838 were rectal swabs and 153 were ETA samples. The pattern of initial bacterial colonization is presented in Fig 1.

*S*. *aureus* was the most commonly isolated organism from nasal swabs, cultured from 110 patients (13.1%): 72/110 (65.5%) of these were MRSA. The rate of nasal methicillin-resistant *Staphylococcus aureus* colonization among patients transferred from other hospitals (9.5%, 48/505) was higher than among those admitted from the community (7.2%, 24/333) but not statistically significant (p = 0.25). From the 740 (88.3%) patients from whom we isolated *E*. *coli* from a rectal swab, 437 (59.1%) were colonized with ESBL-producing *E*. *coli*, 45 (6.1%) with AmpC-producing *E*. *coli* and 8 (1.1%) with carbapenemase-producing *E*. *coli*. *K*. *pneumoniae* was the most commonly isolated organism from tracheal aspirates, isolated from 11.8% (18/153) of patients.

The overall admission prevalence of bacterial isolation from at least one of the body sampling sites with either *S*. *aureus*, *E*. *coli*, *Klebsiellla* spp., *Pseudomonas* spp. or *Acinetobacter* spp. was 93.7% (785/838). The prevalence of bacterial isolation was variable between different patient subgroups: 95.8% (319/333) for subjects originated from the community, 77.6% (392/505) for those being transferred to ICU from other hospitals (p ≤ 0.001, OR = 6.57, 95% CI (3.70–11.67)). The AROs colonization rate at all sites was 63.1% (529/838). 62.2% (207/333) patients admitted from the community were colonized with AROs and that of patients transferred from other hospitals was 63.8% (322/505) (p = 0.64).

### Antimicrobial susceptibility of colonized bacteria on ICU admission

From all sampling swabs, a total of 1,905 colonizing bacterial isolates were isolated: 143 (7.5%) were *S*. *aureus*, 1,370 (71.9%) were *E*. *coli*, 345 (18.1%) were *Klebsiella* spp., 32 (1.7%) were *Pseudomonas* spp. and 15 (0.8%) were *Acinetobacter* spp.. The antimicrobial susceptibility profiles of the isolated organisms are summarized in Table 2. MRSA accounted for 63.6% of all *S*. *aureus* isolates, vancomycin-resistant *S*. *aureus* was not detected. The rate of 3^rd^-generation cephalosporin resistance and ciprofloxacin resistance in *E*. *coli* was approximately 50%. The rate of 3^rd^-generation cephalosporin resistance and ciprofloxacin resistance in *Klebsiella* spp. was approximately 20.0% and 12.5%, respectively. Approximately 10% of *Pseudomonas* spp. were ceftazidime-resistant. Markedly, 60% of the isolated *Acinetobacter* spp. were resistant to imipenem or meropenem. We additionally found that a small proportion of *E*. *coli* and *Klebsiella* spp. (3.6% and 6.4%, respectively) were resistant to colistin, the antimicrobial of last resort for carbapenemase-producing organisms (CPOs).

**Table 2:** Antimicrobial resistance of colonized bacteria on ICU admission.

Antimicrobials n (%)	*S*. *aureus* (n = 143)	*E*. *coli* (n = 1370)	*Klebsiella* spp. (n = 345)	*Acinetobacter* spp. (n = 15)	*Pseudomonas* spp. (n = 32)
**Amoxcillin-clavulanic acid**		746 (54.5)	82 (23.8)		
**Ceftazidime**		**702 (51.2)**	**74 (21.4)**	13 (86.7)	**3 (9.4)**
**Ceftriaxone**		**703 (51.3)**	**76 (22.0)**	13 (86.7)	
**Cefepime**		639 (46.4)	56 (16.2)	12 (80.0)	3 (9.4)
**Ticarcillin-clavulanate**		828 (60.4)	87 (25.2)	12 (80.0)	21 (65.6)
**Piperacillin-tazobactam**		651 (47.5)	51 (14.8)	12 (80.0)	3 (9.4)
**Ofloxacin**		632 (46.1)	31 (9.0)		
**Ciprofloxacin**	**70 (49.0)**	646 (47.2)	43 (12.5)		
**Levofloxacin**	**70 (49.0)**			10 (66.7)	3 (9.4)
**Sulfamethoxazole-trimethoprim**	5 (3.5)	953 (69.6)	106 (30.7)	7 (46.7)	29 (90.6)
**Amikacin**		26 (1.9)	8 (2.3)	6 (40.0)	2 (6.3)
**Gentamycin**					2 (6.3)
**Ertapenem**		**29 (2.1)**	**10 (2.9)**		
**Imipenem**		**13 (0.9)**	**10 (2.9)**	**9 (60.0)**	**6 (18.8)**
**Meropenem**		**15 (1.1)**	**10 (2.9)**	**9 (60.0)**	**7 (21.9)**
**Colistin**		**50 (3.6)**	**22 (6.4)**	0	0
**Penicillin**	137 (95.8)				
**Oxacillin**	**91 (63.6)**				
**Vancomycin**	0				
**Erythromycin**	95 (66.4)				
**Rifampicin**	3 (2.1)				
**Clindamycin**	**89 (62.2)**				

## Discussion

Bacterial colonization of patients on admission to ICUs has been studied in some areas around the world. Data from a cluster-randomized trial in 13 ICUs in 8 European countries (2008–2011) reported a percentage of 13.5% (125/926) of patients colonized with highly resistant *Enterobacteriaceae*, MRSA, and vancomycin-resistant *Enterococcus* (VRE) on admission [10]. In a Turkisk ICU, general admission colonization with either MRSA, VRE, ESBL-producing *E*. *coli*, ESBL-producing *Klebsiella* spp., *P*. *aeruginosa*, or *Acinetobacter* spp. was observed in 56.8% (54/95) of patients: 60.0% (9/15) for patients from the community and 56.3% (45/80) for those from other health services [11]. Our study noted a rate of colonization with AROs of 62.2% (207/333) among patients admitted from the community which was as high as 63.8% (322/505) for those transferred from other hospitals. Our setting is perhaps unique in that a large proportion of patients are admitted directly from the community and we have shown a similarly increased rate of AROs in those patients compared to those from other hospitals.

In our study, admission *S*. *aureus* nasal colonization rate was 13.1% which was indeed lower than previously reported in urban and rural northern Vietnam (21.1%, 140/662) [12] and in other countries with rates of between 8.1% and 30.1% [13–16]. However, the prevalence of nasal MRSA colonization of 8.6% (72/838) in this study compared to proportion of MRSA colonization documented in the HTD (2.9% in 2004–2006) [17] and reported in other healthcare settings with rates of between 0.3% and 12.9% [14,18–22] may indicates that MRSA is an increasing problem that needs addressing in Vietnam. So, we do not believe that our microbiological approach resulted in significant reduction in the yield of *S*. *aureus* from the patient’s samples.

There are several limitations of this study that warrant further discussion. This was a single-center study, so, the findings may not be representative in other institutions. However, to our knowledge, due to a lack of data at the country level, our study is the first to analyze colonization, especially with AROs on ICU admission in Vietnam. Furthermore, our unique intake of patients also provides an insight into AROs colonization outside of the hospital setting in a resource-limited setting. However, we are not reporting the genetic mechanisms of antimicrobial resistance to better understand current resistance and evaluate potential interventions.

## Conclusions

Vietnamese ICU patients have a high rate of colonization with AROs and are thus at risk of subsequent infections with these organisms if good infection control practices are not in place. For many of these organisms, there are limited treatment options available, therefore further research to better understand the relationship between colonization and infection and evaluate new prevention strategies is urgently needed.
